# Initial Specimen Diversion Devices for Emergency Department Blood Cultures May Reduce Contamination Rates and Costs: Structured Literature Review

**DOI:** 10.5811/westjem.50723

**Published:** 2026-07-16

**Authors:** Makayla D. Ayres, Maria C. Johnson, Megan E. Andersen, Alyson P. Lewis, Lucas S. Farrow, Amelia M. Vopat, Kathleen M. Spears

**Affiliations:** * University of Missouri-Kansas City School of Medicine, St. Joseph, Missouri; † Mosaic Life Care, St. Joseph, Missouri

## Abstract

**Introduction:**

Emergency departments (ED) are a primary site of blood culture collection in the United States. High rates of blood culture contamination, commonly seen in EDs, are associated with diagnostic inaccuracy, unnecessary antibiotic use, and increased costs. In this structured literature review, we evaluated the effectiveness of initial specimen diversion devices (ISSDs), which are attached to the needle tip and discard the initial 0.15–1.5 mL of blood. We paid particular attention to their applicability in reducing blood culture contamination in adult ED patients in rural healthcare settings. The average cost of an ISDD is $15–$30, and this upfront cost creates a significant barrier in rural EDs, which face resource scarcity.

**Methods:**

In this review of primary literature from 2021–2025, we evaluated the efficacy of ISDD in lowering blood culture contamination rates in adult patients in the ED. Our primary outcome was the change in contamination rates after introduction of the ISDD. Secondary outcomes included changes in vancomycin duration of therapy, hospital length of stay, and total hospital costs.

**Results:**

We screened 460 records across PubMed, EBSCO, and Embase. Eight papers were selected for full review, from 10 hospital systems. All studies demonstrated a decrease in the rates of blood culture contamination with the ISDD. Contamination rates were reduced by an average of 2.89% across all 10 study locations. Only three of 10 hospitals (30%) achieved a blood culture contamination rate below 1%. One rural community hospital saw an absolute reduction of BCCs from 3.94% to 1.05%. Another study reported a decrease in total yearly hospital costs from $1,120,000 to $383,690, and another reported a 31.4% reduction in mean duration of vancomycin therapy.

**Conclusion:**

This review shows the clinical value of ISDDs to reduce blood culture contamination, and highlights the need for further research into scalable implementation strategies, especially in resource-constrained systems, like rural hospitals.

## INTRODUCTION

Blood culture contaminations contribute to healthcare waste and adverse patient outcomes including delayed targeted therapy, higher patient costs, and increased length of stay in the hospital.^1^ Following blood culture contamination, the patient often receives an unnecessary course of antibiotics.^2^ Depending on the route of administration, antibiotic courses may require the patient to remain in the hospital to receive treatment, putting them at higher risk for hospital-acquired infections that further compound length of stay.^2^ Additionally, as the patient receives antibiotics for a blood culture contamination, they are at risk for medication side effects such as acute kidney injury and associated infection with *Clostridium difficile*.^2^

A cost analysis performed in 2019 at the University of Houston demonstrated an approximately $4,500 increase in hospital-associated costs for patients who had a contaminated blood culture sample while inpatient.^3^ The same study also identified increased length of stay by two days, attributable to blood culture contamination.^3^ A second study indicated an increase of hospital costs of up to approximately $5,800 due to such contamination.^4^ Because of the potential for adverse effects on patients, the Clinical Laboratory Standards Institute (CLSI) announced a new recommendation in 2022 to maintain blood culture contamination rates below 1%.^5^

A blood culture is contaminated if it demonstrates growth of bacteria known to colonize skin. Specific species considered to be contaminants vary based on institution but are widely accepted to be from the genuses *Staphylococcus, Corynebacterium*, and *Micrococcus*. In prior work we investigated protocol weaknesses that lead to blood culture contamination and proposed minimally invasive quality improvement (QI) measures to offset these effects. Educational campaigns were implemented to promote adherence to blood culture collection protocol, which demonstrated successful reduction of contamination in the short term.^6^ A limitation of this study and of other related QI initiatives exists in the sustainability of change, necessitating investigation into more sustainable interventions that bypass human error in the blood culture collection process.

Prior studies have proposed solutions to the nationally elevated rates of blood culture contamination through use of initial specimen diversion devices.^2^,^7^–^13^ These small, single-use devices siphon the first predetermined amount of blood from a blood culture sample to prevent contamination. The device attaches to the distal end of the blood draw needle and connects the circuit to the collection bottle with the intent of removing bacteria introduced into the sample through initial skin penetration. The central hypothesis of this device rests on the understanding that the initial aliquot of blood taken in a blood culture sample is most likely to contain skin surface contaminants.^13^

The average cost of a single device is $15–$30 based on manufacturer and distributor.^1^,^3^,^10^ Trials of initial specimen diversion devices have been reported over the past 15 years and demonstrate efficacy in reducing blood culture contamination at their respective institutions.^14^,^15^ Emergency departments (ED) are particularly inundated with elevated rates of blood culture contamination due to the fast-paced environment and sheer volume of samples taken when compared to other hospital departments.^1^ Furthermore, rural EDs face unique challenges regarding resource scarcity, underscoring the importance of implementing fail-safe devices to reduce patient costs and optimize outcomes. Rural hospitals function on lower operating margins compared to urban hospitals (3.1% compared to 5.4%, respectively).^16^ Therefore, use of such devices in the ED of rural hospitals could confer great results in terms of offsetting cost and in patient outcomes as they relate to blood culture contamination. Our objective in this structured literature review was to assess the efficacy and generalizability of initial specimen diversion devices as they relate to utilization in EDs and their potential impact in rural settings.

**Population Health Research Capsule**

What do we already know about this issue?

*ED blood culture contamination increases costs, drives unnecessary antibiotics, and worsens patient outcomes. ISDDs show promise in reducing contamination*.

What was the research question?

*Do initial specimen diversion devices reduce blood culture contamination in adult ED patients, including in rural settings?*

What was the major finding of the study?

*Diversion devices lowered ED contamination rates by an average 2.89% across 10 hospitals, with some sites achieving <1%*.

How does this improve population health?

*Reducing contamination decreases unnecessary antibiotics, lowers hospital costs, and may especially benefit resource-limited rural EDs*.

## METHODS

### Search Criteria

We conducted a literature search via databases including PubMed, EBSCO, and Embase databases using the key terms “initial specimen diversion device” AND “blood culture” OR “blood culture contamination.” All studies published between 2021 to the present day were considered. We selected studies included in review and formal analysis based on the following criteria: primary research; publication after 2021 (in the post-pandemic era) reported outcomes related to blood culture contamination rates in adult patients in the ED; results with quantitative data; reported completion of initial specimen diversion devices trial, and conduction of the study within the United States. The 2020 Preferred Reporting Items for Systematic Reviews and Meta-Analyses (PRISMA) guidelines were used to screen possible papers for inclusion. For this structured literature review, we defined the primary outcome measure as the change in blood culture contamination rate after implementation of an initial specimen diversion device. We also investigated the secondary outcomes of vancomycin duration of therapy, hospital length of stay, and total hospital costs.

### Statistical Analysis

We used descriptive statistics, primarily through averages, to provide a summary of findings from each paper and the aggregate efficacy of the device trials. Linear regression was used to ascertain the significance between blood culture contamination reduction and device type. Statistical significance was determined with a *P* value < .05.

### Cost Analysis

A study by Buzard et al (Hospital 2) included a cost analysis that compared total hospital cost in adult patients (≥ 18 years of age) undergoing blood culture collection in the ED before and after the implementation of an initial specimen diversion device to estimate potential cost savings.^10^ Total hospital costs include expenditures associated with antibiotic therapy, repeat cultures, length of stay, labor costs, and laboratory testing.

## RESULTS

### PRISMA Flow Diagram

**Figure 1:**
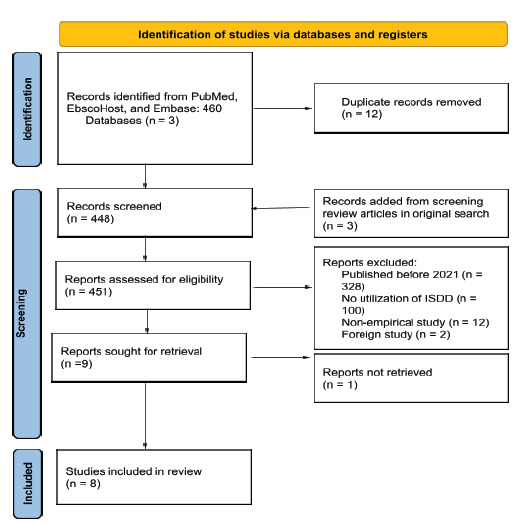
PRISMA flow diagram for paper selection in a study on initial specimen diversion device effectiveness in the emergency department. *ISDD*, initial specimen diversion device; *PRISMA*, Preferred Reporting Items for Systematic reviews and Meta-Analyses.

We followed the 2020 PRISMA guidelines to determine inclusion of studies in this structured literature review (Figure 1). We identified 460 records on initial literature search. After screening records based on the inclusion criteria for this study, nine records were chosen for review. We excluded one record due to limitations in access, as only an abstract had been published. Eight records were included in the structured literature review comprising data from 10 individual institutions.

All studies reported reduction in rates of blood culture contamination after implementing initial specimen diversion devices. Details of each study included in the analysis are summarized in Table 1. Three of the eight studies included data from trials conducted at two separate sites, as denoted by 1a/b, 6a/b, and 7a/b. Trial periods ranged from 5–31 months. Each study included in this review was evaluated for conflicts of interest and funding sources. One study received the devices from Magnolia Medical Technologies, Inc, the manufacturer for the SteriPath initial specimen diversion devices, via a Cooperative Research and Development Agreement.^11^ A second study received funding from a grant through the U.S. Centers for Disease Control and Prevention’s Emory Prevention Epicenter Program.^12^ The remaining studies either received support via their parent hospital institutions,^7^,^9^ declared no conflicts of interest,^2^,^8^ or did not specify altogether.^10^

### Results by Contamination Reduction

Relative reductions in blood culture contamination rates ranged from approximately 21%–100% across study sites, with absolute reductions varying by study design and implementation context.^2^, ^7^–^12^ Arenas et al reported a 100% relative reduction in contamination rates after five months of Steripath use,^8^ indicating elimination of false-positive bacteremia diagnoses during the study period. Results from a second site in the same study using Kurin devices demonstrated a 93.88% relative reduction.^8^ Nielsen et al similarly reported an 89.55% relative reduction following six months of Steripath implementation.^11^

One large multi-year retrospective analysis demonstrated a sustained absolute decrease of 2.1% in blood culture contamination rates over 20 months.^12^ The only rural hospital included in this review achieved a 73% relative reduction in contamination rates and a post-intervention contamination rate of 1.05%.^2^ Across all 10 sites, the mean post-intervention contamination rate was 1.37%,^2^, ^7^–^12^ consistently below the national benchmark of 3%. The pooled average absolute decrease from baseline was 2.89%, with an average relative reduction of 64.16%.^2^,^7^–^12^ Three sites achieved post-intervention blood culture contamination rates below 1%.^8^,^11^

### Results by Device Type

Five sites implemented Steripath initial specimen diversion devices and five implemented Kurin devices. Steripath-based studies demonstrated a greater average absolute reduction in blood culture contamination rates (3.59%)^2^,^7^,^10^–^12^ compared with Kurin-based studies (2.19%).^2^,^8^,^9^ Average relative reductions followed a similar pattern, with Steripath devices associated with a 74.57% relative reduction 2, 7, 10–12 and Kurin devices with a 53.74% relative reduction.^2^,^8^,^9^ These results were not statistically significant (Table 2).

### Results by Length of Stay and Cost

Only one study, reported by Buzard et al (Hospital 2), analyzed the effect of initial specimen diversion devices on length of hospital stay.^10^ They reported no significant difference in length of stay before and after implementing initial specimen diversion devices. However, they did report a significant reduction in total hospital cost from an average of $1,120,000 pre- to $383,690 post-device implementation.^10^ Cost savings from reduced blood culture contamination rates generally arise from avoiding downstream consequences that include unnecessary antibiotic use, repeat testing, additional procedures, and prolonged length of stay.^3^

### Results by Antibiotic Usage

Three hospitals reported further data on vancomycin usage after the implementation of initial specimen diversion devices. Buzard et al (Hospital 2) reported no significant change in vancomycin duration of use following implementation of the devices.^10^ Nielsen et al (Hospital 3), however, did report a significant decrease in vancomycin duration of therapy (DOT) during their study.^11^ They reported an 18.4% decrease in vancomycin DOT with the implementation of nucleic acid amplification testing for eight months prior to the initiation of the initial specimen diversion device trial. After implementation of the trial, they reported another 31.4% decrease in vancomycin DOT.^11^ Wilber et al (Hospital 5) reported an initial increase in vancomycin DOT of 1.1%, followed by a slow trend downward that averaged 0.9% per month over the course of their study.^12^

## DISCUSSION

To the best of our knowledge, this is the first structured literature review of its type to aggregate data investigating the impact of using initial specimen diversion devices exclusively in EDs in the United States in the post-COVID-19 era. Blood cultures are the main tool to identify bacteremia in ED patients and guide further inpatient care. It is imperative to accurately identify causative agents quickly for the best patient outcomes. Blood culture contamination compromises patient safety, increases hospital waste, and increases patient and hospital cost. Based on the findings from this review, use of initial specimen diversion devices demonstrate success in the effective reduction of blood culture contamination rates in adult patients across EDs in 10 different hospitals.^2^,^7^–^12^ All hospital sites demonstrated significant reduction in contamination rates below the previously established goal of < 3%, but only three reported rates < 1% in accordance with the 2021 CLSI guidelines.^2^,^5^,^7^–^12^ While SteriPath devices demonstrated a greater decrease in contaminants in these studies, this work does not intend, nor have the power, to compare device manufactures. Both devices demonstrate meaningful decreases. Additionally, it is important to consider the study duration when assessing the efficacy of these devices to reach the new benchmark.

One study, conducted by Wilber et al, had a study period over one year but only demonstrated blood culture contamination relative rate reduction by 44.98%.^12^ The average study duration was 9.33 months.^2^,^7^–^12^ The three most successful studies, as defined by rates of post-initial specimen diversion devices contamination rates of < 1%, had an average study duration of eight months.^7^,^11^ Due to the relatively small number of included studies, we did not assess. duration of the study as an independent factor influencing the reduction of blood control contamination rates. Follow-up intervals and surveillance should be performed regularly to assess long-term efficacy as these devices become increasingly used in hospital systems.

While initial specimen diversion devices demonstrate efficacy in reducing blood culture contamination rates, their applicability in rural settings requires deeper examination. The implementation of these devices in rural hospitals presents unique challenges as these facilities operate with limited budgets, fewer specialized staff, and constrained infrastructure. Rural hospitals struggle to justify the initial investment in initial specimen diversion devices even when long-term savings and improved patient care is evident. Studies suggest that successful implementation of new practices in rural settings depends on early stakeholder engagement, integration into existing workflows, and external funding support.^17^,^18^

Including rural leadership in planning and evaluation phases may enhance buy-in and sustainability. Despite these limitations, rural hospitals may stand to benefit the most from the use of initial specimen diversion devices due to their heightened vulnerability to the clinical and financial consequences of contaminated cultures. The only study conducted at a regional community hospital in a rural setting reported a 73.35% relative reduction and a 2.89% absolute reduction in blood culture contamination rates from their ED following initial specimen diversion devices implementation. The absence of any studies examining the implementation or impact of these devices in resource-limited rural settings is a significant limitation of the current literature.^2^ Additionally, Buzzard et al discuss that hospitals without rapid diagnostic testing may derive greater benefit from initial specimen diversion devices when compared to hospitals with rapid diagnostic testing for blood culture processing.^10^ In the authors’ experience, it seems rural hospitals function more often without rapid diagnostic testing.

Previous research done by the authors of this review demonstrated marginal improvement of blood culture contamination rates through implementation of educational initiatives alone for healthcare professionals obtaining blood culture samples.^6^ While this has not been directly tested by these authors, it is reasonable to assume that educational initiatives and initial specimen diversion devices, when used in tandem, would further reduce contamination rates in EDs. Sautter et al conducted a systematic review comparing a variety of interventions including initial specimen diversion devices, phlebotomy teams, and educational/training initiatives.^19^ This group reports that regardless of the intervention, high-intensity education and training of staff is more strongly associated with a decrease in blood culture contamination rates than any intervention alone.^19^ Other initiatives such as education on correct blood draw protocol, the use of nucleic acid amplification test for most common causes of sepsis, and specific blood draw teams could help improve contamination rates when coupled with the use of initial specimen diversion devices.^1^,^11^ Further research should investigate the role of multiple modalities to address human error and educational gaps as causative agents for high rates of blood culture contamination.[Fig f2-wjem-27-913]

Implementation of initial specimen diversion devices was also found to be associated with decreased vancomycin use at three hospital sites.^8^,^11^ Antibiotic stewardship is critical to prevent antibiotic resistance. Fewer days of vancomycin use prevents resistance to these potent broad-spectrum antibiotics. However, use of initial specimen diversion devices and reduced blood culture contamination rates were not associated with reduced length of stay for patients.^10^ Length of stay was analyzed by only one study.^10^ Future studies should investigate how blood culture contamination rates and repeat blood culture draws influence vancomycin use, DOT, and length of stay. With these metrics, cost should also be considered to further quantify the financial burden of blood culture contamination rates on both patients and hospital systems.

While initial specimen diversion devices are shown to reduce contamination rates in EDs, more studies of a similar design are needed to analytically demonstrate the efficacy of these devices as a standard of patient care. A 2023 systematic review by Mohajer and Lasco evaluated the efficacy of both brand-name initial specimen diversion devices mentioned earlier, as well as an open technique that requires manual disposal of the first 1 mL of blood taken after collection across multiple hospital departments.^15^ While their results demonstrated significant improvement in blood culture contamination rates with the implementation of initial specimen diversion devices, their study failed to demonstrate a ubiquitous sample of studies reaching contamination rates < 1%.^15^ Callado et al also performed a systematic review of initial specimen diversion devices between 2013–2023 and performed a meta-analysis of three homogenous studies within their study selection.^14^ The results of their meta-analysis suggest a significant reduction in blood culture contamination rates through use of initial specimen diversion devices but no reduction in the detection of true infection in those samples.^14^

## LIMITATIONS

A common limitation described by Mohajer et al and Callado et al lies in the study design and controls for these prospective studies.^14^,^15^ The heterogeneity of the studies encouraged the use of the structured literature review format within this study team, rather than a systematic literature review approach. This review’s specific analysis is limited by the selection criteria in terms of publication date and inclusion of U.S.-only studies. The initial goal was to analyze the effectiveness of initial specimen diversion devices in adult patients in rural EDs; however, only one study met the criteria. This was a regional community hospital in a rural area and likely does not represent resource-limited settings such as critical access hospitals. An expanded search subsequently included diverse populations but kept the focus on these devices implemented in EDs while addressing the potential benefit of implementation in rural hospitals. The availability of data concerning washout periods was limited. Only one study specified the inclusion of a wash-out period, which occurred for an undefined amount of time between the period of initial specimen diversion devices use and standard equipment use.^8^ The blood culture contamination rate during this washout period was not reported.

Rural EDs have specific and unique needs within the U.S. healthcare system. Future studies should consider the implementation of initial specimen diversion devices in under-resourced rural EDs to characterize the impact of these devices in these communities, including cost implications. The general cost per device ranges between $15–$30, depending on the brand and model, which may create a significant obstacle to resource-limited hospitals.^1^,^3^,^10^ A diversion device priced at $20 per unit would yield an estimated cost of $20,000 for every 1,000 blood culture draws. Despite evidence that diversion devices reduce the roughly $4,500–$5,800 in additional costs incurred per contaminated blood culture, the required upfront expenditure of approximately $20,000 constitutes a significant barrier for hospitals operating with constrained resources.^3^,^4^

## CONCLUSION

Results of eight independent analyses of the use of initial specimen diversion devices demonstrate a significant reduction in blood culture contamination rates in adult patients in hospital emergency departments, with associated benefits such as lower costs of care and a shorter duration of antibiotic therapy. While one study is not sufficient to draw conclusions, we hope future researchers explore funding opportunities and stakeholder engagement strategies, as well as assess the long-term impact of using initial specimen diversion devices in rural EDs. Further work is needed to develop scalable models for implementation and consistent results of blood culture contamination rates < 1%.

## Figures and Tables

**Figure 2 f2-wjem-27-913:**
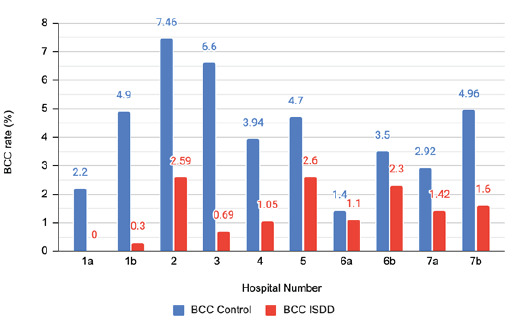
Pre- and post-study blood culture contamination rate change by study site reported in a review of the effectiveness of initial specimen diversion devices in the emergency department. *ISDD*, initial specimen diversion device; *BCC*, blood culture contamination.

**Table 1:** Itemized list of studies included in the review of the effectiveness of initial specimen diversion devices in the emergency department with relevant study details and results. All studies included in this review involved treatment of adult populations.

Hospital number	First author, Year	Setting	Type of study	ISDD type	Study period	Sample size	Absolute BCC change	Relative percentage reduction
1a	Arenas, 2021	Urban	Quality Improvement	Steripath	Nov 2017 – Mar 2018	1,425	−2.2%	100.00
1b				Kurin	Apr 2018 – Feb 2019	2,605	−4.6%	93.88
2	Buzard, 2021	Urban	Retrospective, non-randomized	Steripath	Feb 2018 – Apr 2018; Jun 2019 – Aug 2019	3,331	−4.87%	65.28
3	Nielsen, 2022	Urban	Quality Improvement	Steripath	Oct 2015 – May2016	1,816	−5.91%	89.55
4	Povroznik, 2022	Rural	Prospective, non-randomized	Steripath	Sept 2020 – Apr 2021	3,380	−2.89%	73.35
5	Wilber, 2024	Urban	Quasi-experiment	Steripath	Jan 2018 – Aug 2020	191,789	−2.1%	44.68
6a	Arnaout, 2021	Urban	Prospective, crossover non-randomized	Kurin	Sept 2019 – Feb 2020	1,719	−0.3%	21.43
6b				Kurin	Sept 2019 – Feb 2020	3,942	−1.2%	34.29
7a	Burnie, 2021	Suburban	Quality Improvement	Kurin	Jan 2019 – Jun 2019	Not Reported	−1.5%	51.37
7b				Kurin	Not Reported	Not Reported	−3.36%	67.74

*BCC*, blood culture contamination*; ISDD*, initial specimen diversion device.

**Table 2:** Results of blood culture contamination rate changes stratified by type of initial specimen diversion devices in a scoping review of device effectiveness in the emergency department.

Device type	Average absolute BCC rate change	Average relative BCC percentage reduction	*P* value
Kurin (*n* = 5)	2.19%	−53.74%	.22
SteriPath (*n* = 5)	3.59%	−74.57%	

Of note: dDfferences are not statistically significant.

*BCC*, blood culture contamination.
